# The effects of storage temperature on PBMC gene expression

**DOI:** 10.1186/s12865-016-0144-1

**Published:** 2016-03-15

**Authors:** Jun Yang, Norma Diaz, Joseph Adelsberger, Xueyuan Zhou, Randy Stevens, Adam Rupert, Julia A. Metcalf, Mike Baseler, Christine Barbon, Tomozumi Imamichi, Richard Lempicki, Louis M. Cosentino

**Affiliations:** Leidos Biomedical Research, Inc., Frederick, MD 21702 USA; Division of Clinical Research, National Institute of Allergy and Infectious Diseases (NIAID), National Institutes of Health (NIH), Rockville, MD 20852 USA; Biogen, 125 Broadway, Cambridge, MA 02142 USA

**Keywords:** Cryopreservation, Peripheral blood mononuclear cells, Gene expression

## Abstract

**Background:**

Cryopreservation of peripheral blood mononuclear cells (PBMCs) is a common and essential practice in conducting research. There are different reports in the literature as to whether cryopreserved PBMCs need to only be stored ≤ −150 °C or can be stored for a specified time at −80 °C. Therefore, we performed gene expression analysis on cryopreserved PBMCs stored at both temperatures for 14 months and PBMCs that underwent temperature cycling 104 times between these 2 storage temperatures. Real-time RT-PCR was performed to confirm the involvement of specific genes associated with identified cellular pathways. All cryopreserved/stored samples were compared to freshly isolated PBMCs and between storage conditions.

**Results:**

We identified a total of 1,367 genes whose expression after 14 months of storage was affected >3 fold in PBMCs following isolation, cryopreservation and thawing as compared to freshly isolated PBMC aliquots that did not undergo cryopreservation. Sixty-six of these genes were shared among two or more major stress-related cellular pathways (stress responses, immune activation and cell death). Thirteen genes involved in these pathways were tested by real-time RT-PCR and the results agreed with the corresponding microarray data. There was no significant change on the gene expression if the PBMCs experienced brief but repetitive temperature cycling as compared to those that were constantly kept ≤ −150 °C. However, there were 18 genes identified to be different when PBMCs were stored at −80 °C but did not change when stored < −150 °C. A correlation was also found between the expressions of 2′–5′- oligoadenylate synthetase (OAS2), a known interferon stimulated gene (IFSG), and poor PBMC recovery post-thaw. PBMC recovery and viability were better when the cells were stored ≤ −150 °C as compared to −80 °C.

**Conclusions:**

Not only is the viability and recovery of PBMCs affected during cryopreservation but also their gene expression pattern, as compared to freshly isolated PBMCs. Different storage temperature of PBMCs can activate or suppress different genes, but the cycling between −80 °C and −150 °C did not produce significant alterations in gene expression when compared to PBMCs stored ≤ −150 °C. Further analysis by gene expression of various PBMC processing and cryopreservation procedures is currently underway, as is identifying possible molecular mechanisms.

**Electronic supplementary material:**

The online version of this article (doi:10.1186/s12865-016-0144-1) contains supplementary material, which is available to authorized users.

## Background

Human peripheral blood mononuclear cells (PBMCs) are a critical biological specimen type collected in clinical trials and basic science research. PBMCs are used for the evaluation of various in vitro functional and phenotypic immunological assays, e.g., enzyme-linked immunosorbent spot (ELISPOT) assays [1, 2], proliferation assays [3], flow cytometry [4] and cytometry by time-of-flight (CyTOF) [5] determinations. They also serve as precursors for potential immunotherapy development [6] and are used for biomarker discovery [7, 8] in translational medicine. Many times, PBMC samples need to be collected, processed and cryopreserved at multiple clinical sites. As a result, considerable attention has been given to standardization and/or harmonization [9–11] of the needed assay reagents and assay procedures involved in the testing of these samples that measure the patient’s immune response or status. Testing samples in batches is frequently an additional requirement in order to minimize assay variability; therefore, PBMC samples are often cryopreserved and stored at ultra-low temperatures until they are tested. However, the same level of standardization and consistency given to the pre-analytical factors involved in the storage, shipping and general sample handling of frozen cryopreserved PBMCs has not been applied.

According to best practice [12], in order to maintain viability of PBMCs, they need to be stored below −132 °C, the glass transition temperature of water (GTTW). This is the temperature, at or below, that all biological activity stops [13]. Often, biospecimen storage temperature is chosen based on what type of equipment is available at the location where the biospecimen processing and cryopreservation will be performed. Some facilities, both in the US and internationally, cannot easily get liquid nitrogen for specimen storage below −132 °C. It may also be difficult to provide liquid nitrogen to all facilities because of cost or design restrictions in their physical plant. However, samples from these sites may be deemed necessary for the project, e.g., achieving planned participant enrollment numbers or satisfying a demographic requirement. The use of mechanical −140 °C freezers can be an option, but operating procedures need to be in place to avoid repeatedly warming specimens above the GTTW, especially when accessing the freezer for sample retrieval.

These logistical challenges may make it impossible to have PBMCs stored below −132 °C throughout its entire life at all facilities. Weinberg, et al., reported that storage of cryopreserved PBMCs for ≤ 3 weeks at −70 °C did not affect cell viability, recovery or flow cytometry results [14, 15]. Cryopreserved PBMCs have been reported to be stored up to 1.5 years, in a mechanical −70 °C/-80 °C freezer, depending on container type used, without effecting viable cell recovery [16]. How long specimens are at −70 °C/-80 °C may also be dictated by when the samples will be relocated to the central biobank for long-term storage before they are tested. This type of dual-storage temperature approach may be under-reported in the literature, especially since mechanical −70 °C/-80 °C freezers are common equipment at most clinical trial sites. Regardless, temperature fluctuations can occur when specimens are being retrieved from storage, during storage or when shipped. All of these situations need to be avoided due to their negative affect on the viability and functionality of PBMCs after they have been thawed [2, 17].

Functional differences between freshly isolated PBMCs and cryopreserved/thawed PBMCs have been well summarized and reviewed [18]. These differences have been attributed to reagents or procedures used to isolate and cryopreserve the PBMCs [19–21] as well as how the PBMCs were thawed [22]. Several cytokines have been reported to be spontaneously released from thawed cryopreserved PBMCs from Type 1 diabetic children but not freshly isolated PBMCs [23]. Interferon-γ secretion from PBMCs was one of the highest produced cytokines identified from frozen thawed PBMCs [23, 24]. Likewise, apoptosis and necrosis is detected after cryopreserved PBMCs are thawed [25, 26]. This outcome has been described as cryopreservation-induced delayed-onset cell death (CIDOCD) [27], which accounts for the decrease in PBMC viability and recovery [28].

In this report, we use gene expression analysis to identify several major stress-related cellular pathways and real-time RT-PCR to confirm that specific genes associated with these pathways are affected when cryopreserved PBMC samples are stored for 14 months at different temperatures and then thawed. We also extend our previous observations about how cell viability and recovery are affected by cryopreservation by showing how 2′–5′-oligoadenylate synthetase 2 (OAS2) gene expression in freshly isolated PBMCs inversely correlates with their post-thaw viability. Specifically, gene expression analysis was conducted on fresh and cryopreserved PBMCs under three different storage conditions. Sister aliquots of PBMCs were all control-rate frozen down to −150 °C and then stored: (1) at ≤ −150 °C, which is considered the “gold standard” for storage temperature (2) at ≤ −150 °C for 24 h and then 104 cycles of computer-controlled thermal cycling with repetitive warming to −80 °C and cooling back down to −150 °C; and (3) at −80 °C. Gene expression of cryopreserved PBMCs that were stored at these three storage conditions for 14 months was compared to data from their freshly isolated (non-frozen) PBMC sister samples. In all three storage situations, the gene expression pattern was different from their freshly isolated parent PBMCs. Flow cytometry and viability were also determined from aliquots of the fresh samples and all three storage conditions in an attempt to obtain a comprehensive understanding of two pre-analytical factors, storage temperature and storage temperature fluctuations, during routine sample handling. This work provides critical insight into the molecular changes that are occurring within stored PBMCs following hypothermic transition and storage in a frozen state. This work also provides a foundation for establishing scientifically-based “Best Practices” in specimen storage and for identifying biomarkers of quality for cryopreserved PBMC specimens.

## Conclusions

In the present study, we show that total/unfractionated PBMCs that were cryopreserved and stored at <-150 C, <-150 C + Cycled and -80 C for 14 months have different gene expression patterns than their freshly isolated counterpart. These changes tend to be related to two or more major stress-related pathways (stress responses, immune activation and cell death). Of the 1,367 affected genes that showed >3 foldchange, 66 of these genes were shared among these stress-related pathways, which was confirmed by real-time RT PCR for 13 representative genes. PBMCs stored at -80 C for the same period of time had a significantly lower viability post-thaw and had 18 different genes altered than aliquots stored at <-150 C or at <-150 C + Cycled. Specimen handling and storage decisions may influence intended downstream analysis when susing PBMCs. Therefore, being able to measure gene expression of specific genes such as the interferon stimultory gene, OAS2, allowed us to identify a potentially useful biomarker of poor PBMC quality post-thaw regardless of the storage temperature. Overall, our results provide insight into the reason why it is important to store PBMCs below the GTTW, thus assisting in the establishment of "Best Practices."

## Methods

### Blood donation

Blood was collected from 10 healthy donors in 2005 at the Frederick National Laboratory for Cancer Research’s Normal Donor Program. Each donor signed a NIH 2514–1, Consent to Participate in A Clinical Research Study, under Study Number OH99-C-N046. Whole blood was collected in Acid Citrate Dextrose (ACD)-Solution Vacutainers (Becton Dickenson, Franklin Lakes, NJ).

### PBMC isolation

Whole blood was pooled from vacutainers, to create a homogenate mixture prior to PBMC isolation. The pooled whole blood was divided into 25 ml aliquots. Plasma was collected from the whole blood by centrifugation at 450 x g for 20 min at room temperature (RT), and discarded. The cellular fraction of the whole blood was diluted with RPMI 1640 media without L-Glutamine (Quality Biologicals Inc., Gaithersburg, MD) to bring the volume to 35 ml and gently resuspended prior to PBMC isolation. PBMCs were isolated by density centrifugation using Ficoll-Paque™ Plus (GE Healthcare Bio-Sciences Corp., Piscataway, NJ). Thirty-five mls of the diluted cellular whole blood fraction was overlaid onto 15 mls of the Ficoll-Paque Plus and then subjected to 900 x g for 30 min at RT with the centrifuge brake “off.” PBMCs were washed 2X with RPMI 1640 media by centrifugation at 450 x g for 10 min at RT. PBMCs were counted using Trypan Blue (Sigma, St. Louis, MO) exclusion on a hemocytometer and light microscope. Aliquots were segregated for gene expression analysis.

### Control-rate freezing

The remaining PBMCs were placed in RPMI 1640 media without L-Glutamine (RPMI 1640 media) supplemented with 20 % heat-inactivated HyClone™ Fetal Bovine Serum (FBS, Hyclone Laboratories Inc., Logan, UT) and 7.5 % Dimethyl Sulphoxide Hybri-max® (Sigma, St. Louis, MO). 1 mL aliquots of 10 × 10^6^ cells/ml were created and frozen to −150 °C using a CryoMed Freezer (Thermo Electron Corp., Marietta, OH) with the control-rate program (start temperature +4 °C; −1C/min to −4 °C; −25 °C/min to −40 °C; +15 °C/min to −12 °C; −1 °C/min to −40 °C; −10 °C/min to −150 °C; hold at −150 °C for 10 min).

### PBMC storage

PBMCs frozen to −150 °C were then stored under three different conditions. Sister aliquots from each donor were (1) placed in static ≤ −150 °C storage in the vapor phase of a liquid nitrogen freezer, i.e., Stored ≤ −150 °C; (2) cycled between −80 °C and −150 °C to mimic long-term exposure to repository storage & sorting conditions, i.e., Stored ≤ −150 °C + Cycled (start at temperature of −150 °C; increase +10 °C/min to −80 °C; hold at −80 °C for 10 min; decrease −30 °C/min to −150 °C; hold at −150 °C for 10 min; the samples were cycled through this program for 8 h per day for 13 days; final hold at −150 °C was followed by transfer to vapor phase LN2 for the duration of the storage period); and (3) placed in static −80 °C storage, i.e., Stored −80 °C.

### PBMC thawing

After 14 months of storage, samples were then rapidly thawed at 37 °C. The cells were rinsed with RPMI 1640 media to remove residual DMSO, resuspended in RPMI 1640 media supplemented with 10 % heat-inactivated FBS, and counted by Trypan Blue exclusion for total count and viability. Gene expression analysis was given preference over flow cytometry analysis when cell recovery was low. PBMCs designated for gene expression analysis were placed in the RLT lysis buffer solution as described in the Microarray Gene Expression Analysis section below, and frozen at −80 °C until batch processed. PBMCs designated for flow cytometry were placed in the RPMI 1640 media with 10 % FBS.

### Flow cytometry of PBMCs

Flow cytometric analysis of ACD-anticoagulated ficoll-hypaque isolated PBMCs was performed on a Becton Dickinson LSRFortessa SORP flow cytometer (BD Biosciences, San Jose, CA) and data analysis was performed with DeNovo FCSexpress v4 flow cytometry analysis software (DeNovo, Los Angeles, CA).

The PBMCs were washed in Annexin binding buffer (BD Pharmingen, San Jose, CA) then stained with CD45 Brilliant Violet 510 (Clone HI30, Biolegend, San Diego, CA). Annexin V (AnnV) PerCP-Cy5.5 (BD Pharmingen, San Jose, CA) and 7-AAD (Invitrogen, Grand Island, NY) to identify apoptosis/necrosis. The cells were gated on FS/SSC and CD45/SSC to identify leukocytes apoptotic cells were identified as AnnV positive/7-AAD negative and necrotic cells were identified as AnnV positive/7-AAD positive.

### Microarray gene expression analysis

PBMCs were prepared for gene expression analysis by removing the media from the cells, rinsing with room temperature phosphate buffered solution pH 7.4 (Quality Biologicals Inc., Gaithersburg, MD) and lysing the cells via resuspension in Buffer RLT (Qiagen, Valencia, CA). The lysed cell mixture was stored at −80 °C. Total RNA was isolated from PBMCs that underwent different storage conditions using Qiagen RNeasy kit by following manufacturer’s protocol. Total RNA was quantitated and qualified in Nanodrop 8000 and Agilent Bioanalyzer RNA Nano 6000 chip before performing Affymetrix GeneChip assays. Affymetrix Human GeneChip U133 Plus 2.0 Array (Affymetrix, Sacramento, CA) was used and labeling assay was performed by following manufacturer’s protocol.

Microarray data was normalized and analyzed using Partek Pro (St. Louis, MO). Genes were selected by 3-way ANOVA statistical analysis and correlated with cell viability flow cytometry data. The selection criteria are *P*-value less than 0.05 and absolute fold change more than 3. The correlation selection criteria are *p*-value is less than 0.05 and inter-quartile value is more than 1.4. Selected genes were normalized by medium shift before hierarchy clustering. Biological function annotation was done on in-house software DAVID [29] (http://david.abcc.ncifcrf.gov/) and Ingenuity IPA software (Qiagen, Germantown, MD).

### Quantitative real-time reverse transcription PCR (RT-PCR) assay

cDNA was synthesized from isolated total RNA (600 ng from the same sample for microarray assay; a sample of nuclease-free water was included as RT control) with TaqMan Reverse transcription Kit following manufacturer-provided protocol (Applied Biosystems, Grand Island, NY). Briefly, RNA was mix with 1 μL oligo d(T)_16_ (50 mM in the kit) and then brought to a total volume of 9.6 μL with nuclease-free water. The mixture was homogenized and pelleted by brief vortex and centrifugation. Next, the mixture was incubated at 65 °C for 5 min and then rested at 4 °C for 2 min. After the initial treatment, 10.4 μL prepared master mix was added to each sample. The master mix was composed of the following reagents that were all included in the kit: 2 μL 10 × RT buffer, 1.4 μL 25 mM MgCl_2_, 4 μL 10 mM dNTP mix (2.5 mM each), 1 μL 100 mM DTT, 1 μL RNase inhibitor (20 U/ μL) and 1 μL MultiScribe RT (50 U/μL). The samples were then homogenized and pelleted by brief vortex and centrifugation. Next, the reaction was carried out at 37 °C for 30 min and then deactivated at 95 °C for 5 min. After the deactivation, the samples were set at 4 °C. Before quantitative PCR, the volume of synthesized cDNA was brought up to 120 μL with nuclease-free water. Each sample was run in triplicate wells with 2 μL synthesized cDNA in each well (a sample of nuclease-free water was also included as QPCR blank control). 23 μL prepared QPCR master mix [12.5 μL 2 × TaqMan Universal PCR Master Mix (Applied Biosystems, Grand Island, NY), 1.25 μL 20× human GAPDH primer and probe (Applied Biosystems, Grand Island, NY; VIC-TAMRA-labeled probe), 1.25 μL 20× target gene primer and probe (Applied Biosystems, Grand Island, NY; FAM-labeled probe) and 8 μL nuclease-free water for each sample] was added to each well, homogenized and pelleted by brief vortex and centrifugation. The reaction was carried on Applied Biosystems QuantStudio 12K Flex platform (Applied Biosystems, Grand Island, NY) following the conditions: 50 °C × 2 min (1×), 95 °C × 10 min (1×), followed by 40 cycles of 95 °C × 15 s and 60 °C × 1 min. After the QPCR reaction, the cycle number was obtained by manually setting the threshold of GAPDH and target gene at 0.1 for all samples. The cycle numbers for the same sample were close enough in triplicate wells to guarantee the least variation of operation. Before summarizing the results, the expression of target gene was normalized to the expression of GAPDH in the same well.

## Results

### Cryopreservation greatly impacts the PBMC transcriptome

A microarray cryopreservation study was performed with a total of 39 PBMC samples from 10 healthy donors that were evaluated after fresh isolation and following storage for 14 months. All aliquots of PBMCs were rate-controlled frozen and then handled and stored in one of the following 3 ways before being evaluated for gene expression: (1.) Stored ≤ −150 °C, (2.) Stored ≤ −150 °C + Cycled and (3.) Stored −80 °C, (see “PBMC storage” in Materials and Methods section for complete details). Table 1 summarizes the number of significantly modulated genes for each storage temperature and cycling, if performed. Table 1 indicates that storage of PBMCs under any of three conditions results in changes in a large number of transcripts compared to freshly isolated PBMCs. This is in contrast with the much lower number of transcripts when comparing Stored ≤ −150 °C to Stored ≤ −150 °C ± Cycled (no difference in transcript levels) or Stored −80 °C (18–225 genes, depending on the fold-change threshold). From herein, a fold-change cut-off of >3 fold (1,367 unique genes in total; Additional file 1.xlsx) was used in order to better manage the biological interpretation of gene list as related to the magnitude of the response. Of interest is the effect that cryopreservation has on the magnitude of decreasing the expression of certain genes as well increasing gene expression.

**Table 1:** Number of genes significantly modulated by different storage conditions

Comparison	mRNA level change	Number of genes (FC > 2)	Number of genes (FC > 3)
Stored < −150 °C vs. Fresh	Increased	1453	450
	Decreased	1624	427
Stored ≤ −150 °C + Cycled vs. Fresh	Increased	1507	674
	Decreased	1721	420
Stored −80 °C vs. Fresh	Increased	1134	517
	Decreased	2168	480
Stored ≤ −150 °C + Cycled vs. Stored ≤ −150 °C	Increased	0	0
	Decreased	0	0
Stored −80 °C vs Stored ≤ −150 °C	Increased	56	8
	Decreased	169	10

This table illustrates the number of genes with significantly (*p* < 0.05) higher or lower transcript levels following cryopreservation and thawing as compared to freshly isolated PBMCs or between storage conditions using an absolute Fold-Change cut-off of >2 (FC2) or >3 (FC3)

### Transcriptome profiles of cryopreserved PBMC are associated with immune activation, stress response, and cell death pathways

**Fig. 1:**
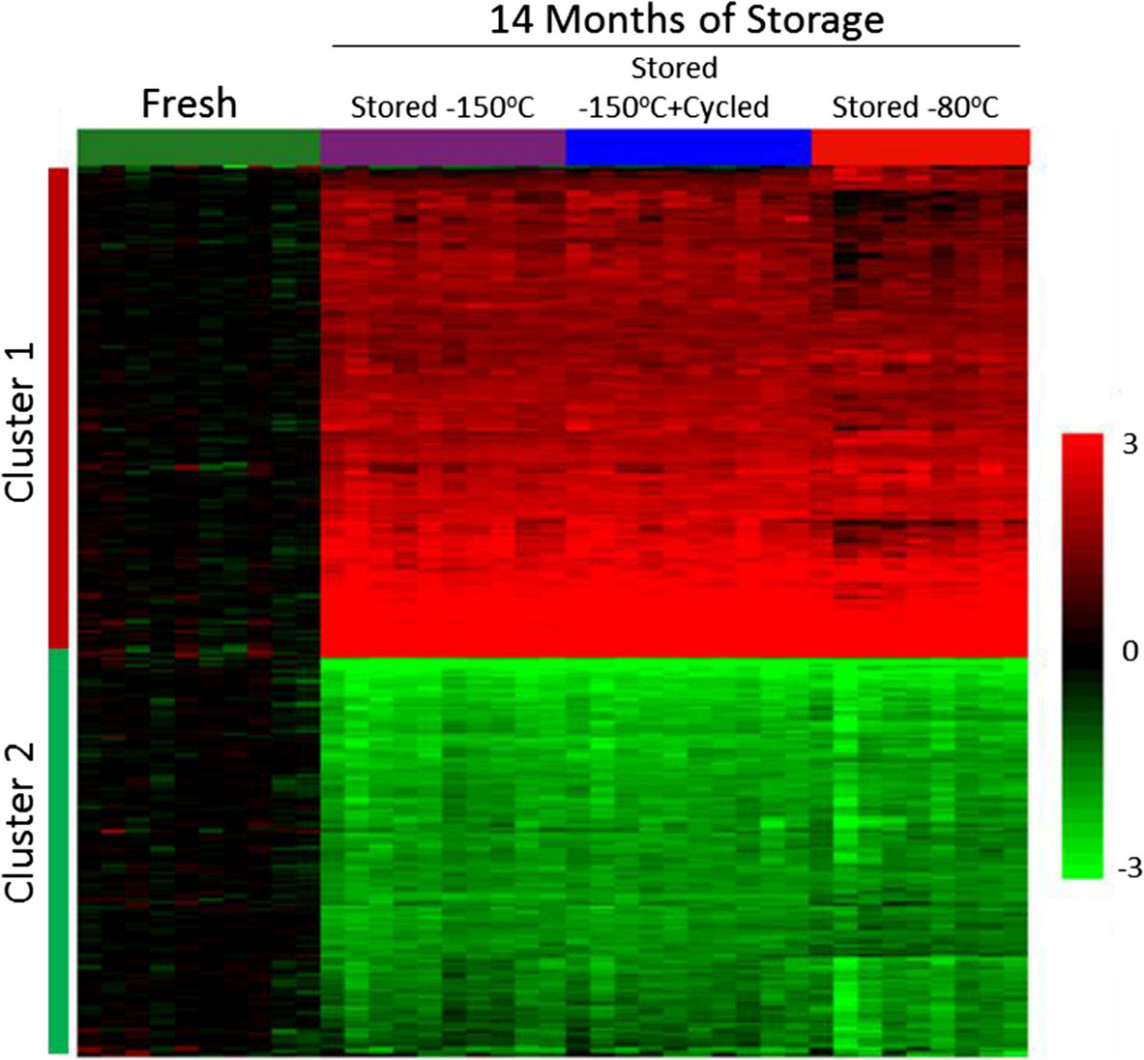
Visual summary of 1,367 differentially expressed genes following cryopreservation. The expressed genes from PBMC samples stored for 14 months in the 3 storage conditions are presented in Cluster 1 (increased expression) and Cluster 2 (decreased expression) as related to freshly isolated PBMCs that have an absolute FC > 3

K-means clustering and heat map visualization were used to summarize gene expression changes of the 1,367 selected genes relative to the median expression value of a given gene within the “Fresh” samples (Fig. 1). The data distributed into 2 major clusters, with Cluster 1 representing 753 genes with higher transcript levels on average by cryopreservation compared to “Fresh” samples, and Cluster 2 representing 614 genes with lower transcript levels compared to “Fresh”.

**Fig. 2:**
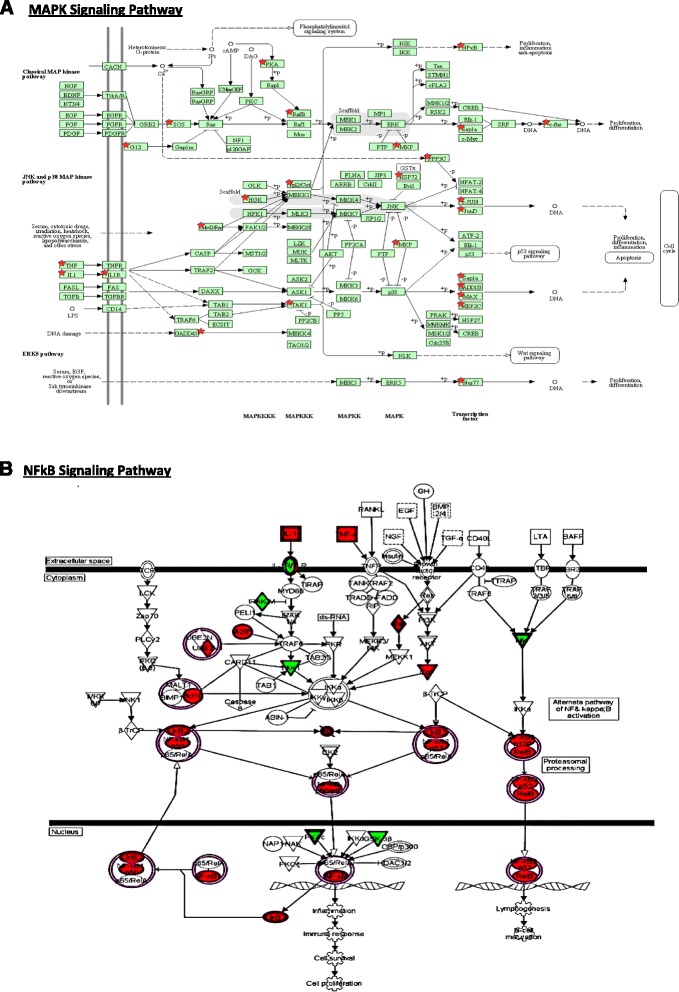
Select Biological Pathways Significantly Associated with Differentially Regulated Genes. MAP Kinase Pathway (**a**) and NFKB Pathway (**b**) are illustrated separately. The selected 1376 transcripts were overlaid in the KEGG MAPK signal pathway through DAVID analysis. The genes in the selected list were marked with red starts. The same list of genes was overlaid on NF-kB signaling pathway through Ingenuity Pathway Analysis software. The red color genes were genes in Cluster 1 (up-regulated by Cryopreservation) and the green color genes were the ones in Cluster 2 (Down-regulated by Cryopreservation)

DAVID and Ingenuity Pathway (IPA) tools were used to annotate and characterize the biological pathways most associated with genes from Cluster 1 and 2. A select list of biological pathways enriched with transcripts was significantly altered following cryopreservation and are shown in Table 2. Additional file 2.xlsx contains supplemental information to Table 2 and contains the list of genes associated with each of the biological pathways. Toll-like Receptor Signaling, Regulation of Transcription, NFkB Signaling, MAP Kinase Signaling Pathways are associated with the set of elevated transcripts (Cluster 1) whereas Cell Death and Defense Response Pathways were similarly associated with both Clusters 1 and 2. Figure 2 shows two pathways: the Kegg Map Kinase Pathway and the IPA NFkB Signaling Pathway targeted by the selected genes.

**Table 2:** Select biological categories significantly associated with differentially regulated genes

Pathway Tool	Database	Pathway	Enrichment *P*-value	Cluster	Number of genes (% of Genes)
IPA	Canonical Pathways	Toll-like Receptor Signaling	4.70E-09	1	12 (16.7 %)
				2	7 (9.7 %)
DAVID	GO-BP	Cell Death	5.80E-08	1	46 (10.6 %)
				2	34 (9.4 %)
DAVID	GO-BP	Regulation of Transcription	9.90E-08	1	141 (32.3 %)
				2	70 (19.4 %)
IPA	Canonical Pathways	NFkB Signaling	1.20E-06	1	17 (10.1 %)
				2	9 (5.3 %)
DAVID	GO-BP	Defense Response	1.50E-05	1	36 (8.2 %)
				2	27 (7.5 %)
DAVID	KEGG	MAPK Signaling Pathway	7.4E-04	1	27 (14.2 %)
				2	7 (5.0 %)

This table summarizes the number genes associated with the common pathways identified by IPA and DAVID that were either in cluster 1 or 2

There were 66 genes out of 212 unique genes shared between 2 or more biological pathways, which are shown in Additional file 3. This observation suggests a strong inter-connection between these pathways as a result of the cryopreservation procedure. Support for this includes the modulation of early response genes from MAP Kinase pathway such as MAP3K7, MAP3K8, MAP4K4, MAPK6, DUSP2, DUSP4, DUSP5, DUSP7, DUSP8, DUSP10, and PPM1L known to be modulated by a variety of stress related stimuli including oxidative stress, osmotic stress, membrane disruption, and heat or cold shock. Modulation of the MAPK stress response pathway has been shown to modulate a number downstream stress response pathways such as JUN Kinase, cell death and DNA binding/transcription. Many such MAPK kinase downstream regulated genes were modulated in this study including NFKBIZ, NFKBIA, NFKB2, NFKB1, JUND, JUN, GADD45A, GADD45B, FOSL2, FOSL1, FOSB, FOS, ELK4, CASP1, BRAF, BCL3, BAG4, and ARAF. Modulation of many of these genes are linked to regulation of immune response genes such as those seen in this study CCL2, CCL20, CCL7, CCR2, CX3CR1,CXCL16, CXCL2, CXCL3, CXCR2, DEFA1, IFNG, IL1A, IL1B, IL8, JAK2, STAT1, TLR4, TLR5, TLR7, TLR8, TNF. Taken together, these results suggest that the cryopreservation procedure of placing cells in a high serum concentration cryococktail with DMSO, cooling, freezing, storing and thawing modulate mRNAs in pathways involved in cellular stress responses that impact downstream response genes involved in cell death and inflammation.

Thirteen genes that were common to the biological pathways listed in Table 2 and Additional file 3 were analyzed by real-time RT-PCR to confirm the microarray analysis. The RT-PCR was performed on the RNA from the 10 PBMC samples Stored ≤ −150 °C for 14 months and on the 9 RNA samples from the PBMCs Stored −80 °C for 14 months. The Stored < −150 °C + Cycled PBMC RNA samples were not tested since we had shown that cycling did not change the gene expression pattern from that of Stored ≤ −150 °C PBMC RNA samples. This data is presented in Table 3. All 13 genes have a good agreement between the real-time RT-PCR data and the microarray data at both temperatures. These 13 genes are involved in the 4 major biological pathways that were listed in Table 2, i.e., Interferon Signature, Cell Death-Related, Immune Regulatory, and MAPK. The expression of 5 of the genes was shown to be decreased and the expression other 8 genes was increased.

**Table 3:** Comparison of microarray data to RT-PCR data for 13 genes that align with biological pathways

Associated biological pathway	Tested gene^a^	Microarray data (Log2[FC])		RT-PCR data (ΔΔCT)	
		Stored ≤ −150 °C to Fresh	Stored −80 °C to Fresh	Stored ≤ −150 °C to Fresh	Stored −80 °C to Fresh
Interferon Signature Genes	MX1	−0.97	−0.88	−1.93	−1.17
	MX2	−0.74	−0.50	−1.60	−0.91
	IFNG	2.21	3.22	2.50	4.38
	IFI16	−1.81	−1.47	−2.93	−1.77
	OAS2	−1.79	−1.91	−3.29	−2.49
Cell Death-Related Genes	GADD45B	2.10	2.49	1.48	2.61
	FOSL2	3.14	2.98	2.19	2.49
Immune Response Genes	IL1B	4.39	5.19	5.91	7.26
	TNF	1.39	2.00	1.56	2.94
	TNFSF10	−3.23	−2.72	−3.92	−2.88
MAPK Genes	NFKB1	1.63	1.32	1.13	1.21
	MAP3KB	3.07	3.20	1.93	2.05
	DUSP4	4.59	4.10	5.85	6.07

^a^ *Abbreviations*: *MX1* (myxovirus [influenza virus] resistance 1, interferon-inducible protein p78 [mouse], *MX2* (myxovirus [influenza virus] resistance 2 [mouse], *IFNG* (interferon, gamma), *IFI16* (interferon, gamma-inducible protein 16), OAS2 (2′–5′-oligoadenylate synthetase 2, 69/71 kDa), *GADD45B* (Growth arrest and DNA-damage-inducible, beta), *FOSL2* (FOS-like antigen 2), *IL1B* (interleukin 1, beta), *TNF*(tumor necrosis factor), *TNFSF10* (tumor necrosis factor (ligand) superfamily, member 10), *NFKB1* (nuclear factor of kappa light polypeptide gene enhancer in B-cells 1), *MAP3K8* (mitogen-activated protein kinase kinase kinase 8), and *DUSP4* (dual specificity phosphatase 4)

### Impact of cell storage conditions on cell viability

Trypan Blue was used to determine cell viability of freshly isolated PBMCs from the 10 healthy donors plus aliquots of the PBMCs that were cryopreserved, stored for 14 months at each of the three storage conditions and thawed. The viabilities as determined by Trypan Blue were all ≥92 %. As a comparison, cell viability, apoptosis, and necrosis were also evaluated by flow cytometry following cell surface detection of Annexin A5 (AnnV) and DNA staining with 7-aminoactinomycin D (7-AAD). AnnV binds phosphatidylserine (PS) exposed on the surface of cells undergoing apoptosis or exposed by loss of plasma membrane integrity in necrotic cells. The DNA of necrotic, but not apoptotic or viable cells, is stained by the vital dye 7-AAD which and is excluded by cells with an intact plasma membrane [30].

**Fig. 3:**
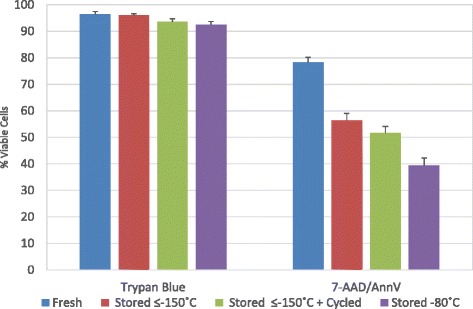
Cell viability differences based on trypan blue and 7-ADD/AnnV. Viability of fresh, ≤ − 150 °C Stored, ≤ − 150 °C + Cycled and −80 °C Stored were determined by trypan blue exclusion and by 7-ADD/AnnV

The percent of live cells as determined by AnnV and 7-AAD staining compared to Trypan Blue varied significantly, both for fresh cells and more so for stored cells (Fig. 3). Cell viability of freshly isolated PBMCs assessed with AnnV and 7-AAD staining averaged 78.5 % +/− 2 %, as oppose to Trypan Blue of 96.3 % +/− 1.1. Cell viability based on 7-AAD/AnnV decreased significantly for all three storage conditions over 14 months as compared to fresh PBMCs, most notably for Stored −80 °C; Stored ≤ −150 °C 56.3 % +/− 2.8 % (*P* < 0.001), Stored ≤ −150 °C + Cycled 51.6 % +/− 2.5 % (*p* < 0.001), and Stored −80 °C 39.4 % +/− 2.8 % (*p* < 0.001). There was no significant difference in cell viability between Stored ≤ −150 °C and Stored ≤ −150 °C + Cycled; however, Stored −80 °C PBMCs had significantly lower viability than PBMCs at Stored ≤ −150 °C (*p* = 0.008). These results are consistent with the microarray data showing no significant gene expression differences between Stored ≤ −150 °C and Stored ≤ −150 °C + Cycling using a 2-fold change threshold whereas 225 genes were significantly different between Stored ≤ −150 °C and Stored −80 °C. However, using a 3-fold change threshold, there were only 18 genes that were different between the PBMCs Stored ≤ −150 °C and those Stored −80 °C. In addition to declining viability during storage, the percentage of cells recovered also declined over time (data not shown). Taking in consideration both cell viability and cell recovery, we find that less than half of the PBMCs put into storage can be recovered as viable cells after 1 year, even under optimal storage conditions of Stored ≤ −150 °C. Interestingly, no such significant decline in cell viability following 14 months of cryopreservation was observed by the use of Trypan Blue indicating the inferiority of this method and how misleading it is.

### Interferon Stimulated Gene (IFSG) levels in fresh PBMC samples predict cryopreservation quality

**Fig. 4:**
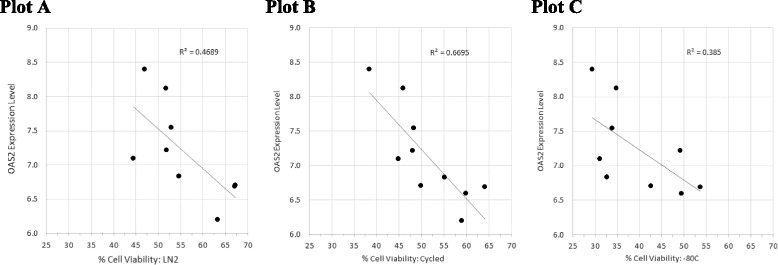
OAS2 gene expression levels in fresh PBMC samples inversely correlates with 14 month cell viability of cells Stored ≤ −150 °C (Plot **a**), Stored ≤ −150 °C + Cycled (Plot **b**) and Stored −80 °C (Plot **c**). *P* values were calculated for each plot: 0.02, 0.002, and 0.037, respectively

Potential biomarkers in fresh PBMC samples that are predictive of cryopreservation quality were identified by selecting genes from fresh samples that significantly correlated with cell viability levels of PBMCs when samples were kept at the three storage conditions. While a number of potential biomarker genes were identified (Additional file 2), a group of interferon stimulated genes were consistently expressed at higher levels in PBMC samples likely to demonstrate poor cell viability in all three storage conditions (Additional file 3). Figure 4 shows an example of one such gene, the 2′5′-oligoadenylate synthetase 2 gene (OAS2).

Each panel in Fig. 4, compares the expression of OAS2 to thawed PBMCs after being cryopreserved and kept at the 3 storage conditions: Plot A (Stored ≤ −150 °C), Plot B (Stored ≤ −150 °C + Cycled) and Plot C (Stored −80 °C). At each storage temperature, the expression of OAS2 was inversely related to the percent cell viability of the PBMCs using 7-AAD/AnnV, which is a further reason not to use or rely on the results with Trypan Blue. The *R* value for each storage condition is presented in Fig. 4. *P* values were also calculated for each storage condition, 0.02, 0.002 and 0.037 respectively, thus demonstrating statistical significance.

## Discussion

Isolating and cryopreserving PBMCs from whole blood is a multistep process. Each step in the process can be variable due to the type and formulation of the reagent used and the temperature and duration of the process performed. Laboratories may have their preference for specific processes and procedures used [18, 19]. Therefore, we have begun a systemic investigation of how various reagents and processes during PBMC isolation and cryopreservation could affect the gene expression of PBMCs in an attempt to establish “Best Practice” recommendations. It is our expectation that this detailed approach will reveal the use of certain reagents or the performance of specific processes that could be critical to identifying biomarkers of specific diseases instead of identifying artificial biomarkers of poor sample handling or storage. Specimen handling and storage decisions could influence intended downstream analysis.

Therefore, in this paper, we evaluated the impact of storing 10 healthy donor cryopreserved PBMCs at the two most common biospecimen storage temperatures (≤ − 150 °C and −80 °C) for 14 months. Even though cell cryopreservation has become prevalent in research and clinical applications since initial experiments demonstrating the feasibility of the technology, and especially since the advent of the use of DMSO cryoprotectants [31, 32], the literature contains reports that differ greatly between these two common storage temperatures or their combination [12–16]. Long-term storage at ≤ −150 °C is considered the “gold standard,” because this is below the glass transition temperature of water (GTTW) [13] and all biological activity is believed to be stopped. If viable and functional cells are required post-thaw, this is the temperature at which published “Best Practices” state cells should be stored [12]. Whereas, storage of PBMCs at −80 °C tends to be for a shorter period of time usually because of resource limitations; however, the literature has reported that it is acceptable for long-term storage as long as the PBMCs are stored in polyvinylchloride or polyolefin bags [16]. However, cryovials of various sizes are the typical storage container for cryopreserved PBMCs and are made of polypropylene. Therefore, it is critical to understand the impact of extended storage time (14 months) on gene expression of cryopreserved PBMCs as related to their viability.

While most conventional cryopreservation methods offer protection of cell membrane integrity during the transition through the hypothermic state to dormancy at the GTTW, cells must contend with multiple other factors that can cause injury and affect viable recovery and function, including temperature fluctuations during storage, shipment and handling [15, 16, 19, 23, 24]. To address the impact that temperature fluctuations may have on cryopreserved PBMCs, we evaluated the effect of controlled temperature fluctuations between −150 °C and −80 °C, e.g., repetitive warming and cooling through the GTTW, which can occur to bystander samples when other sister PBMC-containing vials, are retrieved for testing. We evaluated PBMC viability by two methods: Trypan Blue and AnnV/7-AAD. As previously reported by us [28] and others [33], Trypan Blue evaluation of PBMC viability is not as sensitive as is using flow cytometry using AnnV/7-AAD staining when evaluating apoptosis, delayed apoptosis, or necrotic injury from PBMCs post thawed [19, 20, 24, 25]. These events are underreported when Trypan Blue alone is used to enumerate viable cells [20], and the data in this study recapitulated the phenomenon of inaccuracy of Trypan Blue assessments compared to dual usage of AnnV and 7-AAD.

There was no difference in cell viability using AnnV/7-AAD between PBMCs that were consistently stored at ≤ −150 °C and those stored ≤ −150 °C + Cycled. Likewise, Germann et al. performed a similar experiment and found no change in viability by Trypan Blue but a small decrease in T-cell function (−6.54 % ± 15.89) when cryopreserved PBMCs were cycled under similar temperature parameters and lengths of time at the extreme temperatures [17]. Our current cycling data supports our previous results [34] that it is not the number of brief exposures to a warmer temperature through the GTTW that causes apoptosis/necrosis but rather the length of time of a single exposure. In this paper, the hold time at −80 °C was only 10 min with 104 cycles (17.3 total hours at −80 °C) even though the entire temperature cycling took 52 h to complete. This total length of time was similar to our earlier work [34], which was 48 h but the samples were at −80 °C the entire time, which we believed allowed an increase in apoptosis/necrosis to occur. Taken together, it suggests that it is not the number of cycles or the repetitive temperature changes to −80 °C, nor the accumulative length of time at the warmer temperature, but rather being at the warmer temperature for a protracted period of time. Forty-eight hours was selected because it has relevance to the amount of time that could occur if a specimen shipment is delayed in transit. However, it has been reported that when the temperature inside a cryovial of PBMCs increased to −60 °C, the viability of the PBMCs did decrease even when it was exposed to the warmer temperature for only 5 min [17]. In future work, we plan to determine what the minimum amount of time is, and other parameters needed to cause changes in the highly expressed genes, that are associated with the identified stress-related cellular pathways in cryopreserved PBMCs as described in this paper. This data will help to inform laboratory and biorepository operational decision-making on storage and specimen management.

The analysis of the gene expression patterns showed that all cryopreserved PBMCs, regardless of storage temperature or subjection to temperature fluctuations, were significantly different than its freshly isolated non-cryopreserved parent sample. This difference depended on the fold-change threshold that was chosen to identify affected genes. For this manuscript, we chose a 3 fold-change threshold as our criteria to identify cryopreservation-affected genes of interest. This approach applied criteria of higher stringency for identifying significantly altered genes while not considering gene changes that may be associated with possible background or noise in the systems, and thus that may not be as important. In addition, our preliminary results (data not shown) indicate that culturing stored and thawed cryopreserved PBMCs at 37 °C and 5 % CO_2_ for 24 h modulates thousands of genes but only returns approximately 50 % of storage-modulated genes to baselines levels [35]. This observation will be the focus of future research and publications.

There was a total of 1,367 affected genes that showed a >3 fold-change. The affected genes demonstrated increased or decreased expression and therefore were segregated into 2 clusters, e.g., cluster 1 and cluster 2, respectively. Sixty-six of these genes were shared among two or more major stress-related cellular pathways (stress responses, immune activation and cell death). We also performed real-time RT-PCR and confirmed the involvement in these pathways by interrogating 13 genes that may be involved in at least one of these pathways. Of interest is that there were no genes identified whose transcripts were increased or decreased by >3 fold when the Stored −150 °C PBMCs were compared to the Stored −150 °C + Cycled PBMCs, which underwent cycling between −150 °C and −80 °C. We chose that temperature range because of how rapidly samples that were stored at −150 °C can warm above the GTTW. Our previous work showed that it takes only 4 min for this to occur when samples are being retrieved from a LN2 vapor phase freezer [34]. Recently, Warhurst et al. also showed that samples removed from a LN2 freezer crossed the GTTW within 3 min, which depended on the amount of thermal mass provided by bystander vials that surrounded the thermocouple that was used to measure the temperature. Furthermore, returning samples back into the LN2 vapor freezer took upto several minutes to 3 h in order for the vial to return to its original pre-retrieval storage temperature [36]. As a result, it is critical to better understand what the minimum amount of time needed to cause a change in gene expression in cryopreserved PBMCs is, and does the amount of time need to be an aggregate of multiple smaller time intervals or does it require a single but longer time exposure, as we are suggesting.

In this study, by using microarray analysis we have begun to examine on a molecular level the possible differences between freshly isolated total PBMCs as compared to cryopreserved frozen/thawed total PBMCs. Our data clearly shows there are differences that resulted when PBMCs were subjected to the stress of cryopreservation. Even though, we did not evaluate specific subpopulations of PBMCs, i.e., T cells, B cells, NK cells and monocytes, we cannot dismiss that some of our results may be more aligned with a specific cell phenotype. The focus of this study was to first evaluate total/unfractionated PBMCs, since this is a very common laboratory biospecimen type used when evaluating an immune response. However, future investigations need to explore the potential contribution of the various mononuclear cell subtypes.

In addition, apoptosis and necrosis is well documented to occur during cryopreservation [25–28]. Our microarray data identified their involvement in one of the major stress pathways and our AnnV/7AAD data indicated a decrease in viability that occurred after the PBMCs were thawed. However, our real-time RT-PCR data indicates that apoptosis and necrosis cannot account for all of our gene expression results. As the real-time RT-PCR data has shown there are gene responses (IFI16, OAS2 and TNFSF10) that were decreased but others that were increased (FOSL2, GADD45B, IFI16, IFNG, MAP3KB and TNF) as a result of cryopreservation. Furthermore, there were other genes whose gene expression was enhanced nearly 100-fold (IL1B and DUSP6) and 2 interferon signature genes that were not affected (MX1 and MX2). Taken together, the change in gene expression of some but not all genes may be directly regulated by cell viability. Further analysis is needed to define the mechanism of regulation of those other activated genes involved during the thawing process.

Interestingly, it has been shown that double-stranded breaks (DSB) in DNA can occur in cryopreserved cells with the subsequent induction of DNA repair mechanisms [37]. H2AXγ detection is a highly sensitive measure of DSB formation and this type of genotoxicity is particularly destructive. Phosphorylated H2AXγ formation can occur as a result of cryopreservation and suboptimal storage conditions [37]. Elucidating the degree, types of DNA lesions and extent of DNA repair in PBMC consequent to cryopreservation and storage conditions and length of time in storage are areas of current study in our lab. We have observed double strand DNA breaks as evidenced by positive gH2AX ser139 detection as soon as 30 days post cryopreservation in suboptimally-stored (−80 °C) samples (data not shown).

The induction of H2AXγ in PBMC as a result of cryopreservation and storage is important because it supports the idea that DNA damage is potentially linked to the readily observable increase of apoptosis/necrosis following the thawing of cryopreserved PBMC. This provides an opportunity to interrogate specific molecular-based defensive DNA damage responses.

Determinations of DNA damage and DNA damage responses is rapidly expanding in research related to disease states [38–40] and in the investigation of the specific mechanisms of action of ionizing radiation, viral infections, and chemotherapeutic drugs [41–45] but is currently less understood in cryobiology. There is also an ever-increasing understanding of the mechanisms by which endogenous DNA damage signals can activate innate immune responses, especially effects mediated by increased expression of interferons [46–51]. It has been reported that cryopreserved, as well as irradiated PBMC secrete Type II Interferon (γIFN) [52]. This supports a role for the increase of γIFN expression and in γIFN stimulated gene expression seen in our study. There is considerable cross talk between type I and type II interferon (γIFN), and many IFSG are also TNFα inducible [53]; there was a strong induction of both in our cryopreserved PBMC samples.

**Fig. 5:**
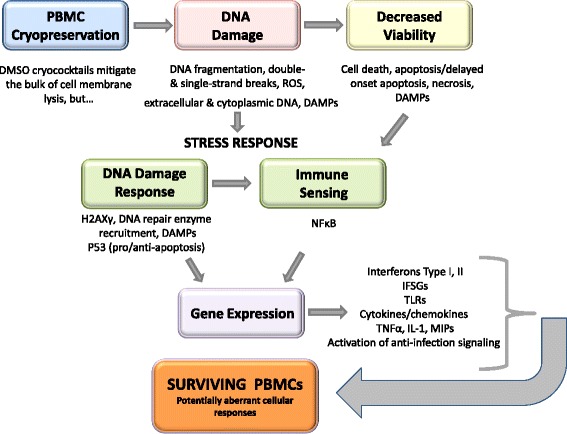
Effects of cryopreservation: An overview

Cell fate relies partially on DNA integrity and cells have evolved the ability to detect genotoxic stress, in order to either induce repair mechanisms or initiate apoptosis to avoid passing on genetic mutations or developing into cancerous cells [44]. Recently, it has been found that PBMC from healthy primates, including humans, can respond to DNA metabolic stress with broad up regulation of multiple Toll-Like Receptor (TLR) genes [50]. As shown in Fig. 5 below, signaling via TLRs upon recognition of pathogen-associated molecular patterns, so called PAMPs, induces γIFN and TNFα and associated cytokines, promoting increased anti-microbial activities as a part of an activated inflammatory response [54]. DNA, whether viral DNA or endogenous damaged DNA, is immunostimulatory. Thus a similarly conserved immune surveillance system includes the evocation of inflammatory and cell defense responses following the detection of cellular damage associated molecular patterns, known as Damage-Associated Molecular Patterns (DAMPs) [49, 50].

Transcript levels of genes in fresh PBMC samples that are correlated with cell viability following cryopreservation may provide insight to pre-storage variables. These correlations may reflect sample cryopreservation quality, such as donor immune state, sample handling and sample processing, and therefore be used as biomarkers of quality. Use of such biomarkers could provide for rapid testing of various handling and processing variables without having to wait long periods of time while samples go through months of storage.

In particular, interferon stimulatory gene (IFSG) levels, i.e., OAS2, in fresh PBMC samples compared to cryopreserved and stored PBMC appeared to predict cryopreservation quality in our study, as these were in good agreement with cell viability. Potential biomarkers in fresh PBMC samples that are predictive of cryopreservation quality were identified by selecting genes from fresh samples that significantly correlated with cell viability levels of cells stored ≤ −150 °C, ≤ − 150 °C + cycled and stored −80 °C. While a number of potential biomarker genes were identified (Additional file 2), a group of interferon stimulated genes were consistently expressed at higher levels in PBMC samples likely to demonstrate poor cell viability in all three storage conditions.

The increased expression of the OAS2 gene appears to be an especially interesting result. This gene product is present in the cytoplasm of cells and promotes foreign nucleic acid degradation, specifically via activation of RNase L and STING (stimulator of interferon genes) [55–57]. OAS2 is also known to be involved in DNA damage/apoptotic responses during sterile inflammation as well [57]. Recently the expression of this gene has been evaluated in several clinical research PBMC biomarker studies. Expression of OAS2 in a triad panel with two other immune response genes was found to be diagnostic of systemic lupus erythematosus (SLE), discriminating between SLE positive subjects and normal controls with 94 % sensitivity [58]. In addition to anti-nuclear antibodies (which can cause DNA damage), and nucleosomes detected in blood, DNA damage in PBMCs has also been reported in SLE patients [40, 59]. Expression of several TLRs and OAS2 in PBMCs was also suggested to play a role in advanced prostate cancer via mechanisms of inflammation and innate immunity [60]. While much more work needs to be done with larger numbers of samples and experimental conditions, OAS2 expression in combination with a select subset of other IFSG in combination with accurate viability measurements, may yield a specific and sensitive quality signature for assessing PBMC samples. IFN-inducible genes, including OAS2 expression, are increased in HIV infected individuals taking antiretroviral treatment and also in elite controllers, consistent with sustained immune system activation despite suppressed plasma viremia [61].

Future work will need to determine if the IFSG biomarkers in fresh PBMCs is a marker of sample handling and processing, or related to the immune state of a donor, although this seems unlikely since the signature associate with OAS2 and expression of other inflammatory mediators in PBMC seems indicative of severe disease, and not healthy donors, as were used in our study. Likewise, careful evaluation of the contribution of the vacutainer type and its anti-coagulant including cell preparation tubes (CPT), wash solutions, cryococktail formulations, method of cryopreservation, e.g., rate-controlled freezer, Mr. Frostie, or CoolCell, and thawing protocol used are all currently being evaluated in the laboratory.

Taken together these data provide promising observations relevant to the continuing development and refinement of a sensitive expression signature in PBMC for assessing cellular damage as a result of cryopreservation. Importantly, these observations reinforce biomarker discovery for PBMC quality at the interface between inflammatory processes, innate immunity and DNA damage cell defense.

### Ethics approval and consent to participate

The specimens were collected under consent as part of clinical research protocol number OH99CN046, Collection and Distribution of Samples from Healthy Donors for In Vitro Research at NCI-Frederick. The protocol was reviewed and approved by the National Cancer Institute Institutional Review Board (NCI-IRB).

## Additional files

Additional file 1: Supplemental data for Table 1. Provides a list of the 1,367 affected genes that demonstrated an increase or decrease in gene expression greater than 3-fold from 14 month cryopreserved PBMCs after they were thawed as compared to fresh PBMCs. It also contains a list of the 18 genes that were affected more than 3-fold when cryopreserved PBMCs were thawed after 14 months of storage at −150 °C versus −80 °C. (XLSX 191 kb) Additional file 2: Supplemental data for Table 2. A list of the 430 genes associated with specific pathways and clusters that demonstrated a greater than 3-fold change as shown in Table 2. (XLSX 31 kb) Additional file 3: Supplemental data showing which genes with a greater than 3-fold change that are affected in multiple pathways. (XLSX 10 kb)

## Abbreviations

DAMPS: damage-associated molecular patterns
DSB: double-stranded breaks
FC: fold change
GTTW: glass transition temperature of water
IFSG: interferon stimulated genes
OAS2: 2′–5′-oligoadenylate synthetase
PAMPS: pathogen-associated molecular patterns
PBMCs: peripheral blood mononuclear cells
γIFN: interferon gamma

## References

[1] Filbert H, Attig S, Bidmon N (2013). Serum-free media support high cell quality and excellent ELISPOT assay performance across a wide variety of different assay protocols. Cancer Immunol Immunother.

[2] Smith J, Joseph H, Green T (2007). Establishing acceptance criteria for cell-mediated-immunity assays using frozen peripheral blood mononuclear cells stored under optimal and suboptimal conditions. Clin Vaccine Immunol.

[3] Aziz N, Margolick J, Detels R (2013). Value of a quality assessment program in optimizing cryopreservation of peripheral blood mononuclear cells in a multicenter study. Clin Vaccine Immunol.

[4] Macchia I, Urbani F, Proietti E (2013). Immune monitoring in cancer vaccine clinical trials: Critical issues of functional flow cytometry-based assays. Biomed Res Int.

[5] Newell E, Sigal N, Bendall S, Nolan G, Davis M (2012). Cytometry by time-of-flight shows combinatorial cytokine expression and virus-specific cell niches within a continuum of CD8+ T cell phenotypes. Immunity.

[6] Kodama A, Tanaka R, Saito M, Ansari A, Tanaka Y (2013). A novel and simple method for generation of human dendritic cells from unfractionated peripheral blood mononuclear cells within 2 days: its application for induction of HIV-1-reactive CD4+ T cells in the hu-PBL SCID mice. Front Microbiol.

[7] Grunblatt E, Bartl J, Zehetmayer S (2009). Gene Expression as peripheral biomarkers for sporadic Alzheimer’s disease. J Alzheimers Dis.

[8] Hindle A, Edwards C, McCaffrey T, Fu S, Brody F (2010). Reactivation of adiponectin expression in obese patients after bariatric surgery. Surg Endosc.

[9] Fox B, Schendel D, Butterfield L (2011). Defining the critical hurdles in cancer immunotherapy. J Transl Med.

[10] van der Burg S, Kalos M, Gouttefangeas C (2011). Harmonization of immune biomarker assays for clinical studies. Sci Transl Med.

[11] Maecker H, McCoy J, Nussenblatt R (2012). Standardizing immunophenotyping for the Human Immunology Project. Nat Rev Immunol.

[12] Campbell L, Betsou F, Leiolani D (2012). 2012 ISBER Best Practices for Repositories: Collection, Storage, Retrieval, and Distribution of Biological Materials for Research. Biopreserv Biobank.

[13] Mazur P (1984). Freezing of living cells: mechanisms and implications. Am J Physiol.

[14] Weinberg A, Song L, Wilkening C (2009). Optimization and limitations of use of cryopreserved peripheral blood mononuclear cells for functional and phenotypic T-cell characterization. Clin Vaccine Immunol.

[15] Weinberg A, Song L, Wilkening C (2010). Optimization of storage and shipment of cryopreserved peripheral blood mononuclear cells from HIV-infected and uninfected individuals for ELISPOT assays. J Immunol Methods.

[16] Valeri C, Pivacek L (1996). Effects of the temperature, the duration of frozen storage, and the freezing container on in vitro measurements in human peripheral blood mononuclear cells. Transfusion.

[17] Germann A, Oh Y-J, Schmidt T, Schön U, Zimmerman H, von Briesen H (2013). Temperature fluctuations during deep temperature cryopreservation reduce PBMC recovery, viability and T-cell function. Cryobiology.

[18] Mallone R, Mannering SI, Brooks-Worrell BM, Durinovic-Bello I, Cilio CM, Wong FS, Schloot NC. Isolation and preservation of peripheral blood mononuclear cells for analysis of islet antigen-reactive T cell responses: position statement of the T-Cell Workshop Committee of the Immunology of Diabetes Society. Clin Exp Immunol. 2010;163:33–49. 10.1111/j.1365-2249.2010.04272.x PMC3010910 20939860

[19] Bull M, Lee D, Stucky J (2007). Defining blood processing parameters for optimal detection of cryopreserved antigen-specific response for HIV vaccine trials. J Immunol Methods.

[20] Afonso G, Scotto M, Renand A (2010). Critical parameters in blood processing for T-cell assays: validation on ELISpot and tetramer plaforms. J Immunol Methods.

[21] McKenna KC, Beatty KM, Vicetti MR, Bilonick RA (2009). Delayed processing of blood increase the frequence of activated CD11b + CD15+ granulocytes which inhibit T cell function. J Immunol Methods.

[22] Ramachandran H, Laux J, Moldovan I, Caspell R, Lehmann PV, Subbramar RA (2012). Optimal thawing of cryopreserved peripheral blood mononuclear cells for use in high-throughput human immune monitoring studies. Cells.

[23] Axelsson S, Faresjo M, Hedman M, Ludvigsson J, Casas R (2008). Cryopreserved peripheral blood mononuclear cells are suitable for the assessment of immunological markers in type 1 diabetic children. Cryobiology.

[24] Mallone R, Martinuzzi E, Blancou P (2007). CD8+ T-cell responses identify beta-cell autoimmunity in human type 1 diabetes. Diabetes.

[25] Fowke K, Behnke J, Hanson C (2000). Apoptosis: A method for evaluating the cryopreservation of whole blood mononuclear cells. J Immunol Methods.

[26] Baust J, Cosentino M, Meeks E (2005). Apoptotic cell death contributes significantly to peripheral blood mononuclear cells cryopreservation failure. Cryobiology.

[27] Baust J (2002). Molecular mechanisms of cellular demise associated with cryopreservation failure. Cell Preserv Technol.

[28] Cosentino M, Corwin W, Baust J (2007). Preliminary Report: Evaluation of storage conditions and cryococktails during peripheral blood mononuclear cell cryopreservation. Cell Preserv Technol.

[29] Huang DW, Sherman BT, Lempicki RA (2009). Systematic and integrative analysis of large gene lists using DAVID Bioinformatics Resources. Nat Protoc.

[30] Zembruski NCL, Stache V, Haefeli WE, Weiss J (2012). 7-Aminoactinomycin D for apoptosis staining in flow cytometry. Anal Biochem.

[31] Lovelock JE, Bishop MWH (1959). Prevention of freezing damage to living cells by dimethyl sulphoxide. Nat Lond.

[32] Fahy GM, MacFarlane DR, Angell CA, Meryman HT (1984). Vitrification as an approach to cryopreservation. Cryobiology.

[33] Mascotti K, McCullough J, Burger SR (2000). Hpc viability measurement: Trypan blue versus acridine orange and propidium iodide. Transfusion.

[34] Lopacznski W, Chung V, Moore T, Guidry J, Merritt L, Shea K, and Cosentino M. Increasing sample storage temperature above −132 °C (glass transition temperature of water [GTTW]) induces apoptosis in cryopreserved human peripheral blood mononuclear cells (PBMCs). International Society of Biological and Environmental Repositories. 2002 Annual Meeting. Poster presentation.

[35] Lempicki RA, Diaz N, Yang J, Adelsberger J, Metcalf JA, Stevens R, Rupert A, Baseler M, and Cosentino LM. PBMC Gene Expression Profiles: Impact of Sample Handling. Society of Cryobiology. 2013 Annual Meeting. Oral presentation.

[36] Warhurst J, Fink J, Holmes T, Albert M and Zandi B. Protection of innocents: Continued sample warm up after return to a cryogenic environment (below −150 °C) following a transient ambient picking operation. International Society of Cellular Therapy. 2015 Conference. Oral presentation.

[37] Peng L, Wang S, Yin S, Li C, Li Z, Wang S, Liu Q (2008). Autophosphorylation of H2AX in a cell-specific frozen dependent way. Cryobiology.

[38] Porcedda P, Turinetto V, Brusco A, Cavalieri S, Lantelme E, Orlando L, Ricardi U, Amoroso A, Gregori D and Giachino C. A Rapid Flow Cytometry Test Based on Histone H2AX Phosphorylation for the Sensitive and Specific Diagnosis of Ataxia Telangiectasia. Cytometry. 2008;73A:508,516. 10.1002/cyto.a.20566 18431795

[39] Kuroi K, Tanaka C, Toi M (2001). Clinical significance of plasma nucleosome levels in cancer patients. Int J Oncol.

[40] Montalvo TM, Miranda-Vilela AL, Roll MM, Grisolia CK, Santos-Neto L (2012). DNA damage levels in systemic lupus erythematosus patients with low disease activity: An evaluation by comet assay. Adv Biosci Biotechnol.

[41] Mariotti LG, Pirovano G, Savage KI, Ghita M, Ottolenghi A, Prise KM, Schettino G. Use of the γ-H2AX assay to investigate DNA repair dynamics following multiple radiation exposures. PLoS One. 2013;8:79541. 10.1371/journal.pone.0079541 PMC3843657 24312182

[42] Tanaka T, Halicka DH, Traganos F, Seiter K, Darzynkiewicz Z (2007). Induction of ATM activation, histone H2AX phosphorylation and apoptosis by etoposide: Relation to cell cycle phase. Cell Cycle.

[43] Kaina B (2003). DNA damage-triggered apoptosis: critical role of DNA repair, double-strand breaks, cell proliferation and signaling. Biochem Pharmacol.

[44] Xiong GM, Gasser S, Vengrova S (2011). Integration of the DNA Damage Response with Innate Immune Pathways. DNA Repair and Human Health.

[45] Mboko WP, Mounce BC, Wood BM, Kulinski JM, Corbett JA, Tarakanova VL (2012). Coordinate regulation of DNA damage and type I interferon responses imposes an antiviral State that attenuates gamma herpesvirus Type 68 replication in primary macrophages. J Virol.

[46] Brzostek-Racine S, Gordon C, Van Scoy S, Reich NC (2011). The DNA damage response induces interferon. J Immunol.

[47] Harberts E, Gaspari AA (2013). TLR signaling and DNA repair: Are they associated?. J Investig Dermatol.

[48] Hӓrtlova A, Erttmann SF, Raffi FAM, Schmalz AM, Resch U, Anugula S, Lienenklaus S, Nilsson LM, Kröger A, Nilsson JA, Ek T, Weiss S, Gekara NO. DNA damage primes the type I interferon system via the cytosolic DNA sensor STING to promote anti-microbial innate immunity. Immunity. 2015;42:332–43. 10.1016/j.immuni.2015.01.012 25692705

[49] Paludan SR, Bowie AG (2013). Immune sensing of DNA. Immunity.

[50] Menendez D, Shatz M, Azzam K, Garantziotis S, Fessler MB, Resnick MA (2011). The toll-like receptor gene family is integrated into human DNA damage and p53 networks. PLoS Genet.

[51] El-Saghire H, Thierens H, Monsieurs P, Michaux A, Vandevoorde C, Baatout S (2013). Gene set enrichment analysis highlights different gene expression profiles in whole blood samples X-irradiated with low and high doses. Int J Radiat Biol.

[52] Venkataraman M (1995). Effects of cryopreservation on immune responses. VIII. Enhanced secretion of interferon-gamma by frozen human peripheral blood mononuclear cells. Cryobiology.

[53] Schroder K, Hertzog PJ, Ravasi T, Hume DA (2004). Interferon γ: an overview of signals, mechanisms and functions. J Leukoc Biol.

[54] Ghosh TK, Mickelson DJ, Solberg JC, Lipson KE, Inglefield JR, Alkan SS (2007). TLR–TLR cross talk in human PBMC resulting in synergistic and antagonistic regulation of type-1 and 2 interferons, IL-12 and TNF-α. Int Immunopharmacol.

[55] Hornung V, Hartmann R, Ablasser A, Hopfner KP (2014). OAS proteins and cGAS: unifying concepts in sensing and responding to cytosolic nucleic acids. Nat Rev Immunol.

[56] Mozzi A, Pontremoli C, Forni D, Clerici M, Pozzoli U, Bresolin N, Cagliani R, Sironi M. OASes and STING: Adaptive evolution in concert. Genome Biol Evol. 2015;7:1016–32. 10.1093/gbe/evv046 PMC4419793 25752600

[57] Dupuis-Maurin V, Brinza L, Baguet J, Plantamura E, Schicklin S, Chambion S, Macari C, Tomkowiak M, Deniaud E, Leverrier Y, Marvel J, Michallet MC. Overexpression of the transcription factor Sp1 activates the OAS-RNAse L-RIG-I pathway. PLoS One. 2015;10:1–19. 10.1371/journal.pone.0118551 PMC4349862 25738304

[58] Grammatikos AP, Kyttaris VC, Kis-Toth K, Fitzgerald LM, Devlin A, Finnell MD, Tsokos GC. A T cell gene expression panel for the diagnosis and monitoring of disease activity in patients with systemic lupus erythematosus. Clin Immunol. 2014;150:192–200. 10.1016/j.clim.2013.12.002 PMC3932542 24434273

[59] Williams RC, Malone CC, Meyers C, Decker P, Muller S (2001). Detection of nucleosome particles in serum and plasma from patients with Systemic Lupus Erythematosus using monoclonal antibody 4H7. J Rheumatol.

[60] Kazma R, Mefford JA, Cheng I, Plummer SJ, Levin AM (2012). Association of the Innate Immunity and Inflammation Pathway with Advanced Prostate Cancer Risk. PLoS One.

[61] Krishnan S, Wilson MP, Sheikh V, Rupert A, Mendoza D, Yang J, Lempicki R, Migueles SA, Sereti I. Evidence of innate immune system activation in HIV type-I-infected elite controllers. J Infect Dis. 2014;209:931–9. 10.1093/infdis/jit581 PMC3935475 24185941

